# Supplementary figures and images for: Multi-State Structural Genomics Enables Large-Scale, Mechanistic, and Context-Specific Classification of ABCC6 Genetic Variants Implicated in Calcification Diseases
Source: Int J Mol Sci. 2026 Feb 14;27(4):1832. doi: 10.3390/ijms27041832 (PMC12940408; doi:10.3390/ijms27041832)

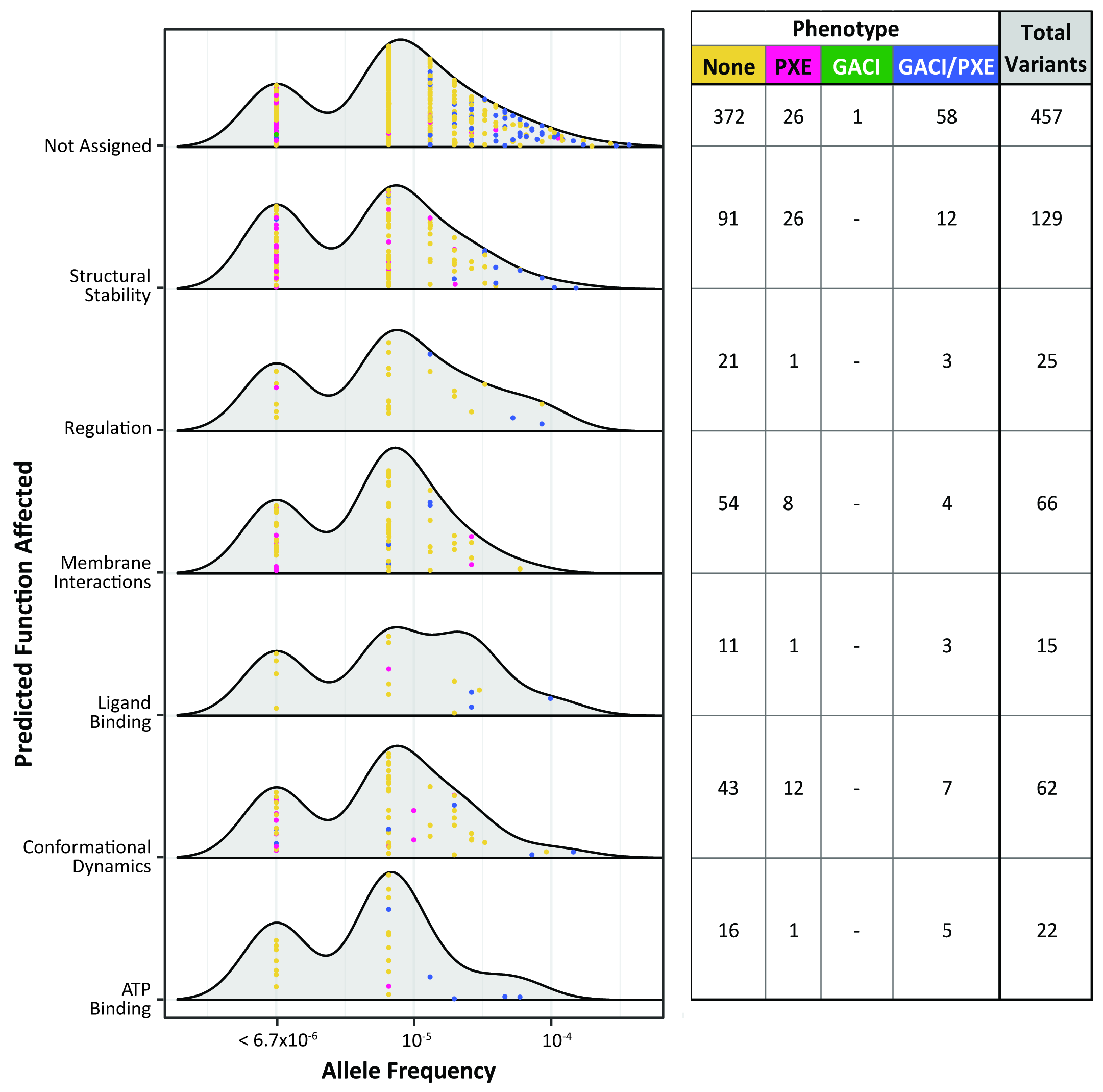

Supplement: Supplementary file 1 [file ijms-27-01832-s001.zip › FigureS2.tif]

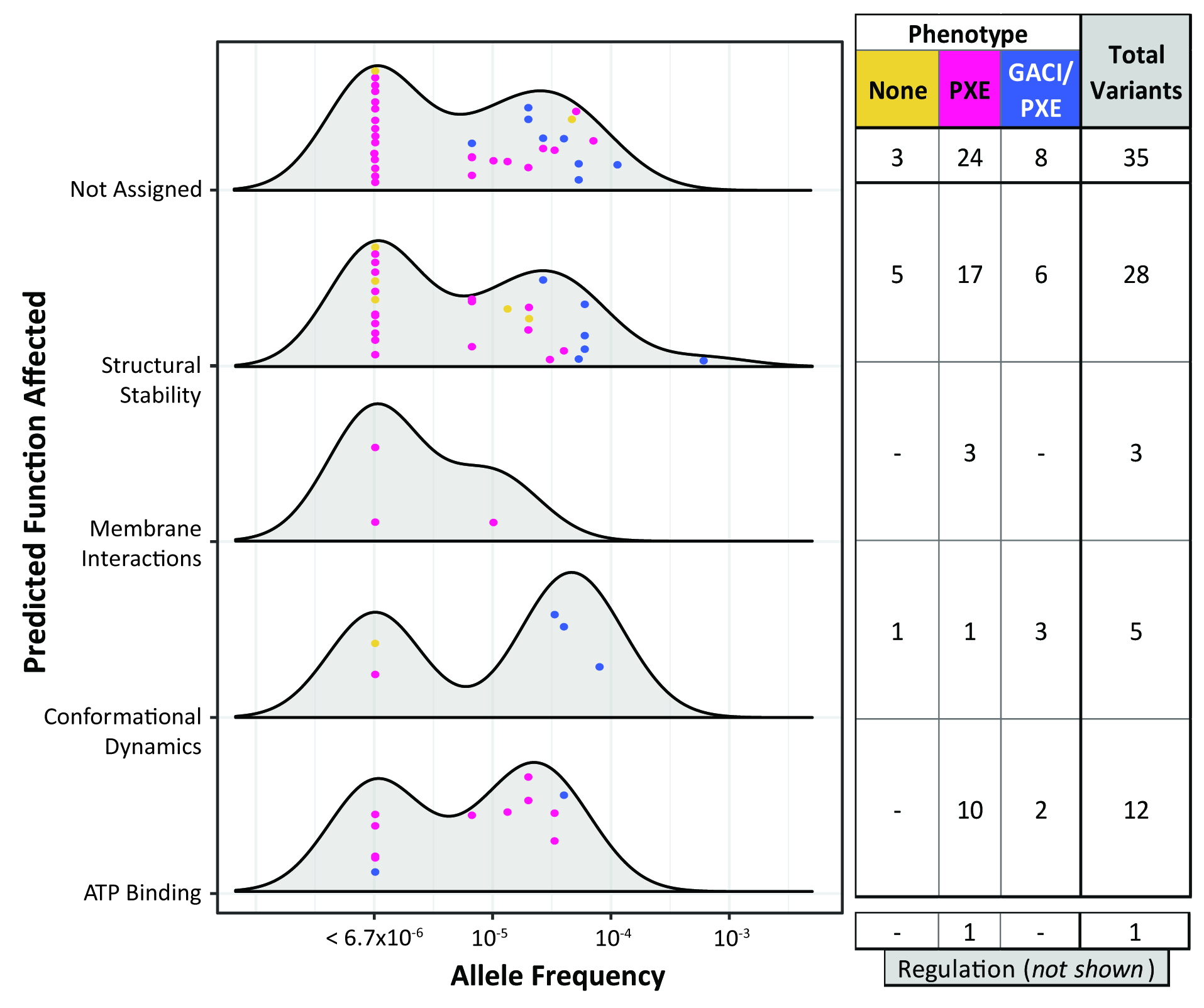

Supplement: Supplementary file 1 [file ijms-27-01832-s001.zip › FigureS1.tif]
